# The cuneus vein: initial anatomical description and preservation technique for posterior interhemispheric approaches

**DOI:** 10.1007/s00701-026-06867-7

**Published:** 2026-04-18

**Authors:** Muhammet Enes Gurses, Abuzer Gungor, Meric Ulgen, Elif Gökalp, Gokberk Erol, Hatice Türe, Uğur Türe

**Affiliations:** 1 Department of Neurosurgery, Yeditepe University School of Medicine, Koşuyolu Mah. Koşuyolu Cad. No: 168, 34718 Kadıkoy, Istanbul, Türkiye; 2 Department of Neurosurgery, Tatvan State Hospital, Bitlis, Türkiye; 3 Department of Neurosurgery, Istinye University, Istanbul, Türkiye; 4 Department of Anesthesiology and Reanimation, Yeditepe University School of Medicine, Istanbul, Türkiye

**Keywords:** Cuneus, Interhemispheric, Microneurosurgery, Posterior, Vein

## Abstract

**Background and objective:**

The posterior interhemispheric approach provides optimal access to lesions located in a number of areas. A major advantage of this approach is that the parieto-occipital vein is typically the only venous structure encountered, which minimizes venous interference during exposure. In a surgical series of 12 patients, we identified a previously undescribed venous variant extending from the cuneus to the falx cerebri. Here, we characterize this venous structure and propose a microsurgical strategy to preserve it.

**Methods:**

A total of 303 posterior interhemispheric approaches were performed from September 2005 to August 2025. We retrospectively reviewed clinical and radiological data collected from patients’ electronic medical records and searched the operative database to locate surgical videos.

**Results:**

In 12 patients undergoing the posterior interhemispheric approach (12/303, 4%), we identified a previously unreported venous variation in which a cortical vein extended from the cuneus to the falx cerebri. We have termed this structure the *cuneus vein*. Among these 12, 2 were observed during a right-sided approach (2/157, 1.3%) and 10 during a left-sided approach (10/146, 6.8%). The vein-releasing technique was used in 7 patients, while the falx-cutting technique was done in 4. In all but one patient, the cuneus vein was successfully preserved. In one patient with hydrocephalus, excessive brain relaxation after CSF drainage limited mobilization and caused vein injury, without hemorrhagic or ischemic morbidity during follow-up.

**Conclusions:**

The cuneus vein, a cortical vein extending from the cuneus to the falx cerebri, is an anatomical variation that has not been described previously but requires careful consideration during the posterior interhemispheric approach. The cuneus vein drains the visual cortex; thus, its sacrifice may lead to postoperative complications, including visual deficits secondary to venous infarction. In this study, we identified and characterized this venous variation and demonstrated its preservation using the vein-releasing technique and the falx-cutting technique.

**Supplementary Information:**

The online version contains supplementary material available at 10.1007/s00701-026-06867-7.

## Introduction

The posterior interhemispheric approach (PIA) is traditionally used to remove lesions in a number of areas: the posterior third ventricle, posterior thalamus, pineal region, tectum, posterior cingulate, posterior falx, precuneus, posterior mediobasal temporal region, atrium, medial parieto-occipital region, and splenium [39–41]. Managing lesions in these areas, especially during exposure and resection, is challenging because of the complex structures. The PIA provides access to the deep parafalcine region and requires only minimal retraction for brain and paraventricular structures via a natural plane. With the increasing implementation of intraoperative magnetic resonance imaging (MRI) in recent years, this approach has been used more frequently [6, 24, 26, 33, 34].

The anterior interhemispheric approach often involves bridging veins, whereas the PIA does not. These bridging veins, encountered within the surgical corridor, may restrict the surgeon’s range of motion and impede access to the lesion during the anterior interhemispheric approach, but maintaining their integrity is essential. In the anterior interhemispheric approach, these veins can be preserved by releasing them or modifying the dural incision [17, 32, 38–40]. A comprehensive preoperative evaluation with MRI scans and three-dimensional reconstruction techniques is essential to identify both deep and superficial venous structures and plan the surgical approach accordingly [12].

In contrast to the anterior interhemispheric approach, the only venous structure expected in a PIA is the parieto-occipital vein. This gives the approach a significant advantage as there are no other large veins obstructing the surgical corridor [8, 14, 18]. This vein is located at the superior corner of the craniotomy and does not interfere with the procedure. In some patients, however, we have found an additional cortical vein extending from the cuneus to the falx cerebri, narrowing the surgical corridor.

Drawing on our extensive experience with the PIA, we describe a previously unreported variation in a cortical vein extending from the cuneus to the falx cerebri, along with techniques to preserve it. To the best of our knowledge, this specific venous configuration has not been documented in the literature. In this study, we present our microsurgical experience with 12 cases of this variation managed using the PIA.

## Methods

This study was conducted according to the principles outlined in the Declaration of Helsinki. This study was approved by the Institutional Review Board (IRB) of Yeditepe University Ethic Committee. Additionally, informed consent was obtained from all patients before their inclusion in the study. All patient data were anonymized and kept strictly confidential in accordance with institutional and ethical guidelines.

A retrospective review of 303 PIAs carried out by the senior author (UT) from September 2005 to August 2025 was conducted. Demographic, clinical, radiological, and operative data were collected from electronic medical records. Comprehensive clinical and visual examinations were done before and after surgery. Patients underwent pre- and post-surgery scans with a 3-T magnetic resonance scanner (GE Healthcare, SIGNA Architect, USA). Contrast-enhanced MRI venography scans (slice thickness 0.625 mm) were evaluated for 303 PIAs, specifically focusing on the vein extending from the cuneus to the falx cerebri.

## Surgical technique

**Fig. 1:**
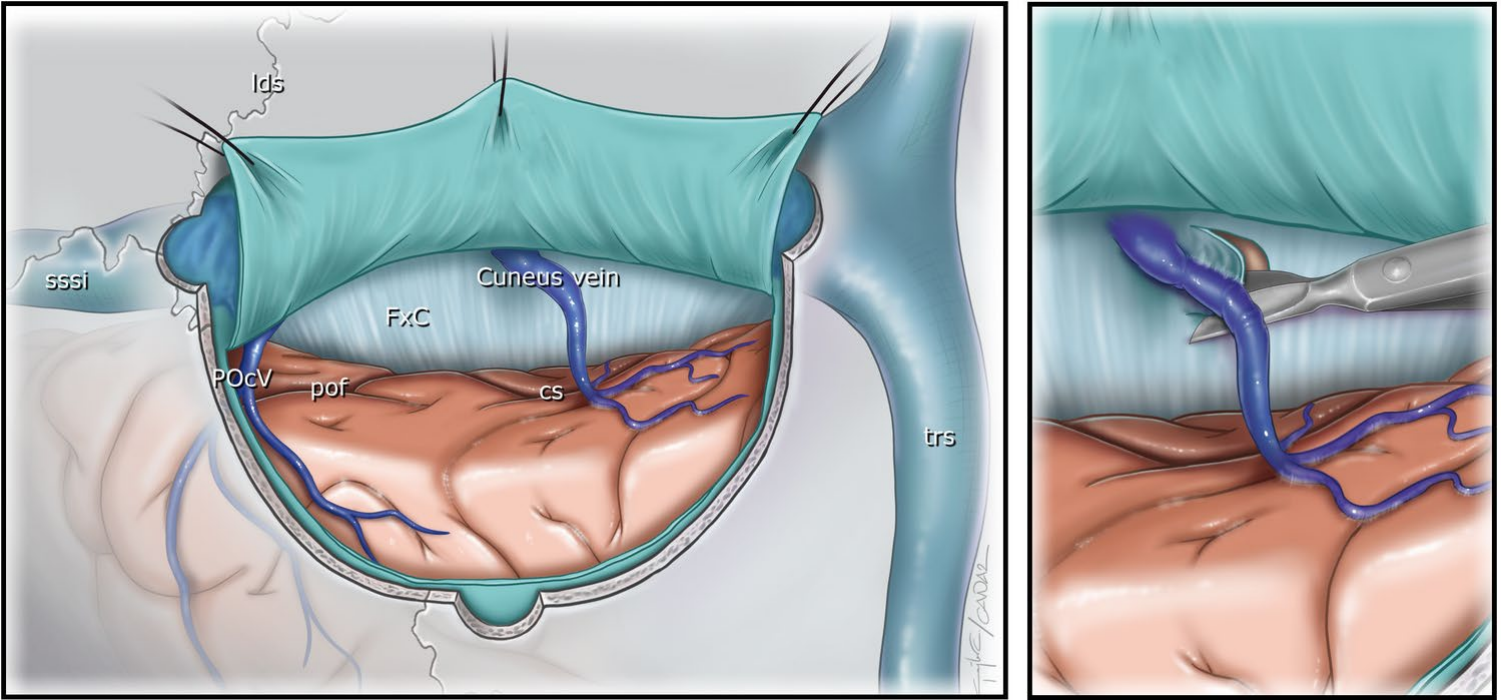
The anatomical course of the typical location and position of the cuneus vein and the falx cutting technique. cs: Calcarine sulcus CV: Cuneus vein FxC: Falx cerebri lds: Lambdoid suture POcV: Parieto-occipital vein pof: Parieto-occipital fissure sssi: Superior sagittal sinus trs: Transverse sinus

All patients underwent a PIA and the deep and superficial venous systems were evaluated with preoperative imaging and three-dimensional reconstruction techniques to guide modifications of the surgical approach as necessary [12]. From 2005 through 2017, surgeries were done with the patient in the lateral oblique position. Once intraoperative MRI was introduced in our clinic in January 2018, procedures were carried out with the patient in the prone oblique position with a 3-pin head holder. The patient’s head was tilted up 15 degrees and rotated downward, orienting the tumor site toward the floor to allow gravity to retract the dependent hemisphere. A horseshoe-shaped incision across the midline was made to access the superior sagittal sinus. After the scalp was retracted, two burr holes were placed over the superior sagittal sinus and one approximately at the lambda. A quadrangular-shaped craniotomy was done and the dura was opened in a semicircle with the base towards the superior sagittal sinus. This step widened the medial operative corridor. Particular attention was given to the vein extending from the cuneus to the falx cerebri. The vein was preserved with the arachnoid-releasing and falx-cutting techniques (Fig. 1, video 1).

## Results

**Fig. 2:**
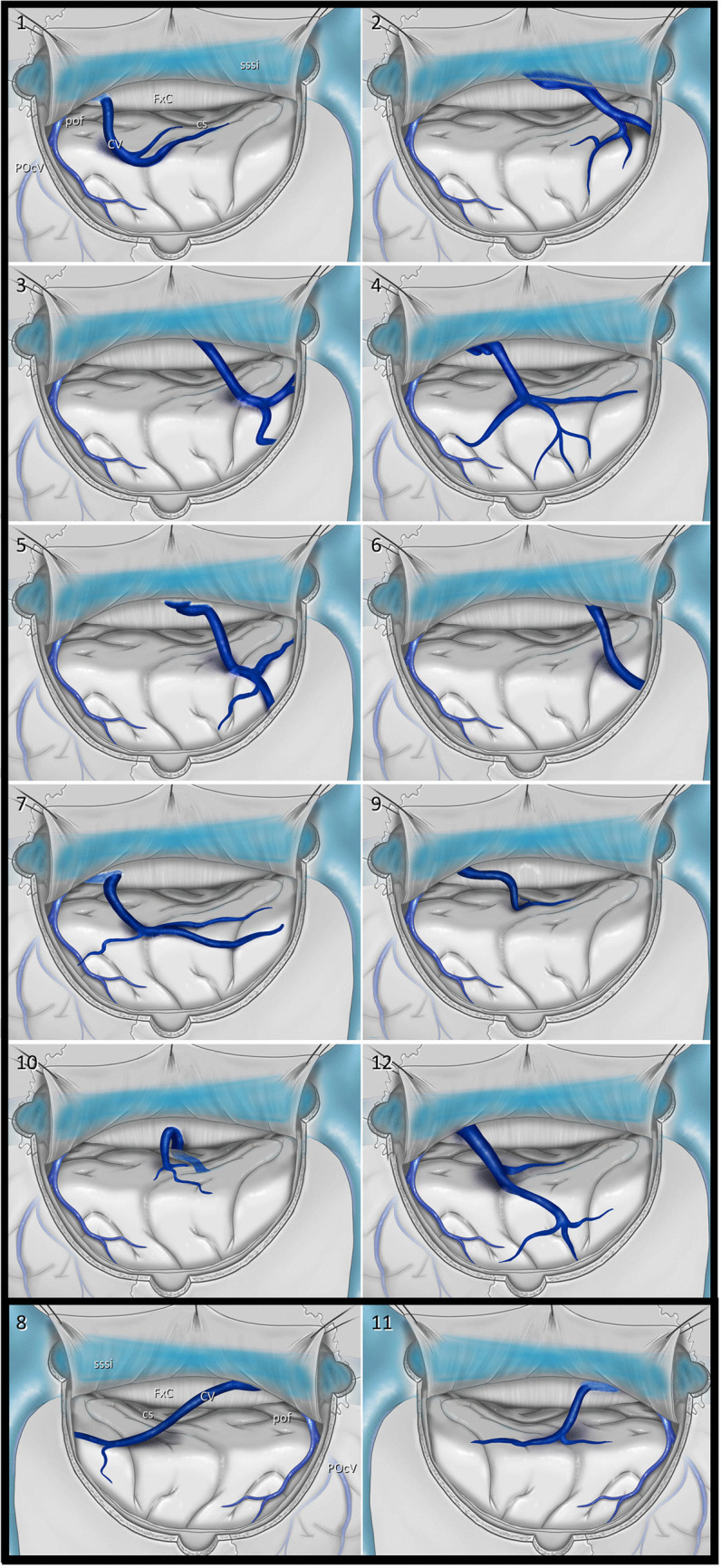
Variations of the cuneus vein in 12 cases: 2 right-sided (cases 8,11) and 10 left-sided (remaining cases). cs: Calcarine sulcus CV: Cuneus vein FxC: Falx cerebri POcV: Parieto-occipital vein pof: Parieto-occipital fissure sssi: Superior sagittal sinus trs: Transverse sinus

Our cohort comprised 303 patients undergoing a PIA. In 12 patients (4%), we identified a venous variation in which a cortical vein extended from the cuneus to the falx cerebri, hereafter referred to as the *cuneus vein* (Fig. 2, Table 1).

**Table 1:** Cases in which the cuneus vein was observed during the posterior interhemispheric approach. The table is arranged in chronological order based on the dates of the cases

Case	Age/Gender	Symptoms	Tumor Location	Side	Postoperative Outcome	Histopathology
1	71/M	Dizziness, bilateral visual field deficit	Thalamus	Left	No neurologic deficit	Epidermal cyst
2	35/M	Generalized tonic–clonic seizure	Mediobasal temporal region	Left	No neurologic deficit	Glioblastoma, IDH-Wild type, WHO Grade IV
3	50/F	Blurred vision	Thalamus	Left	No neurologic deficit	Glioblastoma, IDH-Wild type, WHO Grade IV
4	72/F	Imbalance, headache	Pineal region	Left	No neurologic deficit	Supratentorial ependymoma, WHO Grade II
5	46/F	Tinnitus, headache	Atrium	Left	No neurologic deficit	Meningothelial meningioma, WHO Grade I
6	32/M	Diplopia	Thalamus	Left	No new neurologic deficit	Diffuse midline glioma, WHO Grade IV
7	60/M	Incidental	Mediobasal temporal region	Left	No neurologic deficit	Diffuse astrocytoma, IDH-mutant, WHO Grade II
8	13/M	Double vision, headache	Pineal region	Right	Transient diplopia	Pineal germinoma
9	32/F	Blurred vision, headache	Thalamus	Left	No neurologic deficit	Diffuse midline glioma, WHO Grade IV
10	56/F	Memory loss, headache	Thalamus	Left	Right-sided weakness (4/5)	Glioblastoma, IDH-Wild type, WHO Grade IV
11	20/M	Headache	Pineal region	Right	No neurologic deficit	Pineal parenchymal tumor of intermediate differentiation, WHO Grade II
12	43/M	Headache, right-sided numbness	Thalamus	Left	No neurologic deficit	Glioblastoma, IDH-Wild type, WHO Grade IV

*F* Female, *M* Male, *WHO* World Health Organization

**Fig. 3:**
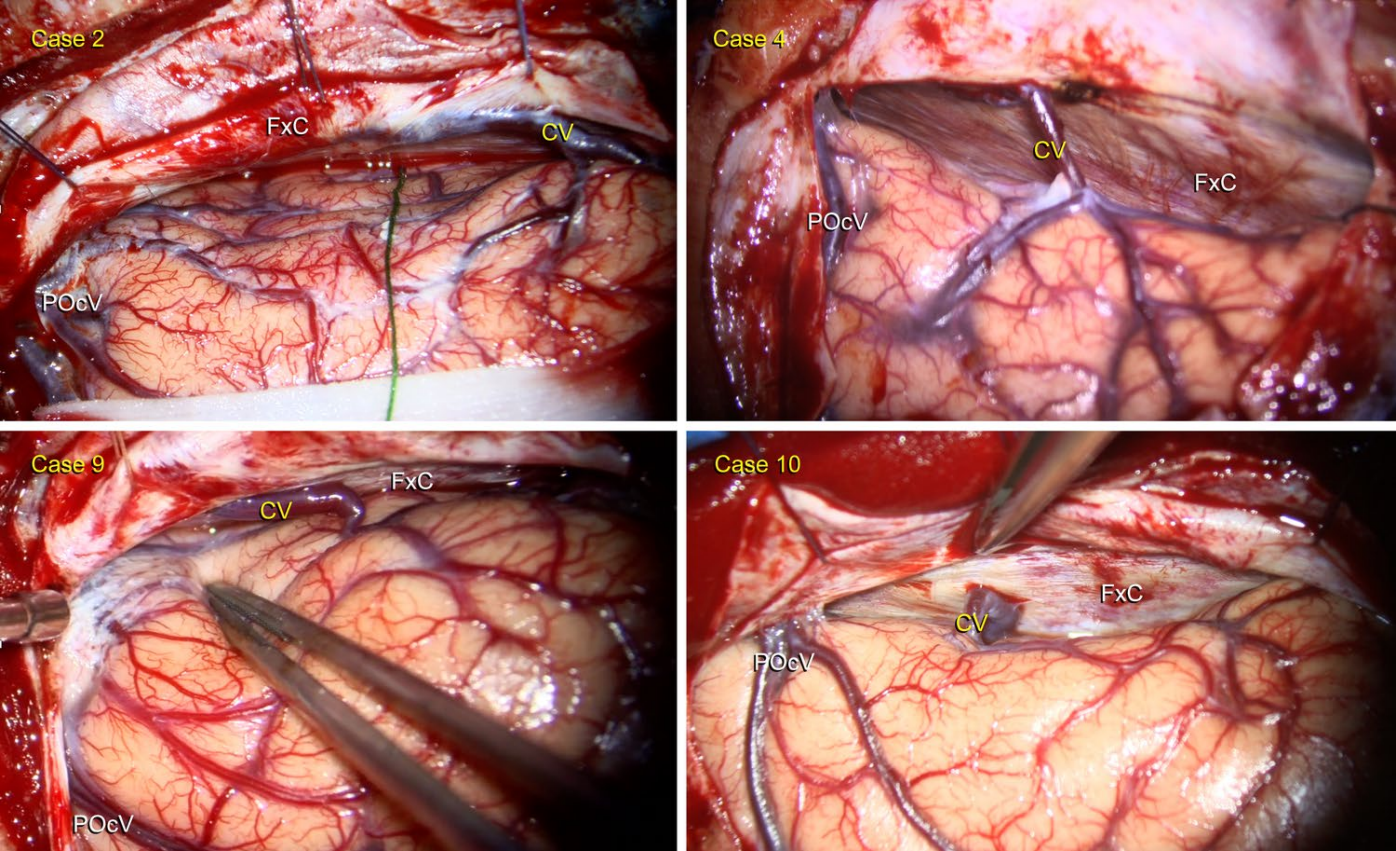
Intraoperative visualization of the cuneus vein in left-sided cases. **A** Case 2**:** A patient with a low-grade glioma in the mediobasal temporal region. **B** Case 4: A patient with a supratentorial ependymoma, WHO Grade II, involving the pineal region. **C** Case 9: A patient with a diffuse midline glioma, WHO Grade IV, with thalamic involvement. **D** Case 10: A patient with a glioblastoma, IDH wild type, WHO Grade IV. CV: Cuneus vein FxC: Falx cerebri POcV: Parieto-occipital vein

**Fig. 4:**
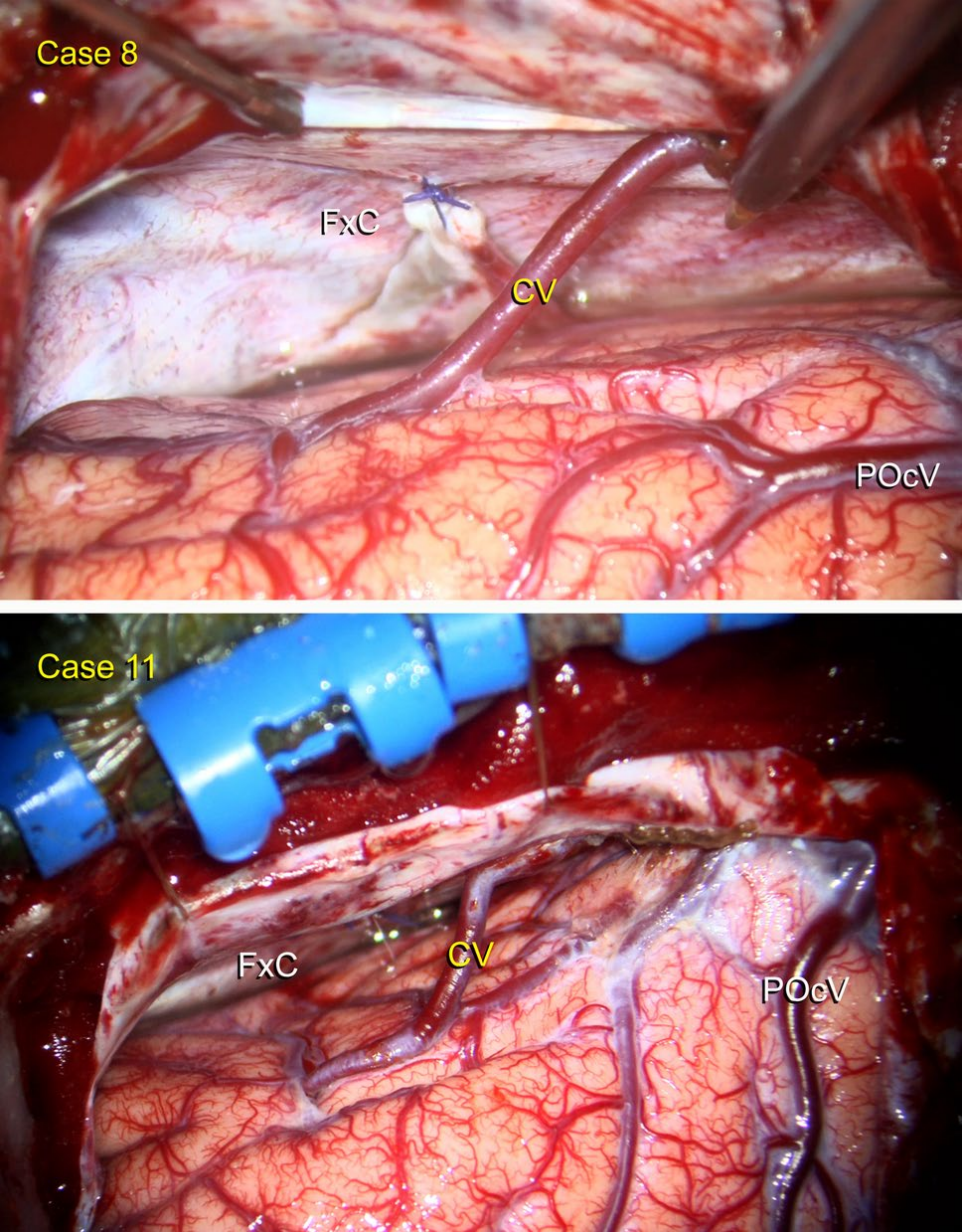
Intraoperative visualization of the cuneus vein in right-sided cases. **A** Case 8: A patient with a pineal germinoma. **B** Case 11: A patient with a pineal parenchymal tumor of intermediate differentiation, WHO Grade II. CV: Cuneus vein FxC: Falx cerebri POcV: Parieto-occipital vein

This variation was observed in 10 left-sided approaches (10/146, 6.8%) (Cases 1–7,9,10,12, Fig. 3) and 2 right-sided approaches (2/157, 1.3%) (Cases 8 and 11, Fig. 4).

In 11 patients, this vein drained blood from the cuneus and coursed from posterior to anterior and inferior to superior directions (Fig. 2). In only one patient it followed a horizontal trajectory from posterior to anterior, draining into the straight sinus via the falx (Case 10, Fig. 3D, Video 1). We used the vein-releasing technique in 7 patients (Cases 2–8), while the falx-cutting technique was utilized in 4 (Cases 9–12, Video 1). This vein was preserved in all but one patient with the falx-cutting technique or opening of the arachnoid membranes over the cuneus vein. The remaining patient had hydrocephalus and cortical atrophy, and when cerebrospinal fluid was drained, the brain overly relaxed and the vein was torn. Because of a cognitive disorder, the patient was unable to adapt to visual field testing; therefore, the assessment could not be completed, but no evidence of hemorrhage or stroke-related morbidity was found during follow-up. This venous variation was not identifiable on preoperative MRI.

## Discussion

The PIA allows the surgeon access to a number of brain structures via a natural corridor: the posterior third ventricle, posterior thalamus, pineal region, tectum, posterior cingulate, posterior falx, precuneus, posterior mediobasal temporal region, atrium, medial parieto-occipital region and splenium. This versatile approach provides an ample surgical field for the posterior portion of the medial parietal and occipital lobes [6, 24, 26, 33, 34]. It is widely accepted that the only venous structure encountered in the PIA is the parieto-occipital vein [8, 11, 14, 18, 21, 22, 28, 31, 40, 41]. In contrast, the anterior interhemispheric approach is known to include wide variability in the location, trajectory, and drainage patterns of superficial cerebral veins [11, 13, 31]. Identifying these vascular variations preoperatively is especially critical in the parasagittal region, given the close proximity of these vessels to the surgical field. Numerous studies have described the variations of parasagittal bridging veins and a few include techniques to preserve these vessels [9, 17, 18, 38]. Several other studies note that increased venous pressure after the sacrifice of a vein may lead to brain damage during retraction and postoperatively, including edema, venous infarction, and hemorrhage [3, 5–7, 19, 26, 37]. The deep venous anatomy exhibits considerable variability and has been described in detail by Serrano et al. [35] A recently published study by Krogager et al. provides a detailed analysis of variations of the galenic venous system [15]. However, our analysis was intentionally limited to the superficial venous anatomy encountered during the posterior interhemispheric approach, and we did not address the deep venous system.

Our preoperative planning consistently prioritizes protecting cortical veins, and we always aim to ensure their preservation during surgery [6, 26]. To do so, extensive preoperative imaging is essential not only to visualize the deep venous systems and locate the optimal space between the bridging veins to access the interhemispheric fissure but also to identify possible variations in the venous system before surgery. This preparation allows the surgeon to modify the approach preoperatively. Preoperative MR venography was performed in all patients, and three-dimensional reconstructions were generated using OsiriX to delineate venous anatomy [12]. Following intraoperative identification of the cuneus vein, all available preoperative MR venograms were retrospectively reviewed to assess its detectability; however, this variant could not be reliably visualized on routine imaging. The vein courses within the interhemispheric fissure and, given the close apposition of the cortex to the falx cerebri, is likely obscured on standard MRI. As CT venography was not routinely obtained in this cohort, the vein was identified exclusively through intraoperative observation, although improved detection may be achievable with ultra–high-field 7-Tesla MRI.

Despite many variations in bridging veins encountered in the anterior interhemispheric approach, only the parieto-occipital vein is encountered during the PIA [21, 23, 40, 41]. This vein drains both the parietal and occipital regions [21, 23, 40, 41]. However, we encountered a venous variation intraoperatively that we were not able to visualize preoperatively. In this variation, a cortical vein extended from the cuneus directly to the falx cerebri. We defined it as the *cuneus vein*. Initially, we considered naming this variant the occipital vein but this term is already used for a different vein originating from the neck [8, 21, 22, 27]. In addition, because Rhoton and colleagues have referred to the “parieto-occipital vein” as the “occipital vein,” [15, 22] we deemed the term *cuneus vein* to be more appropriate.

**Fig. 5:**
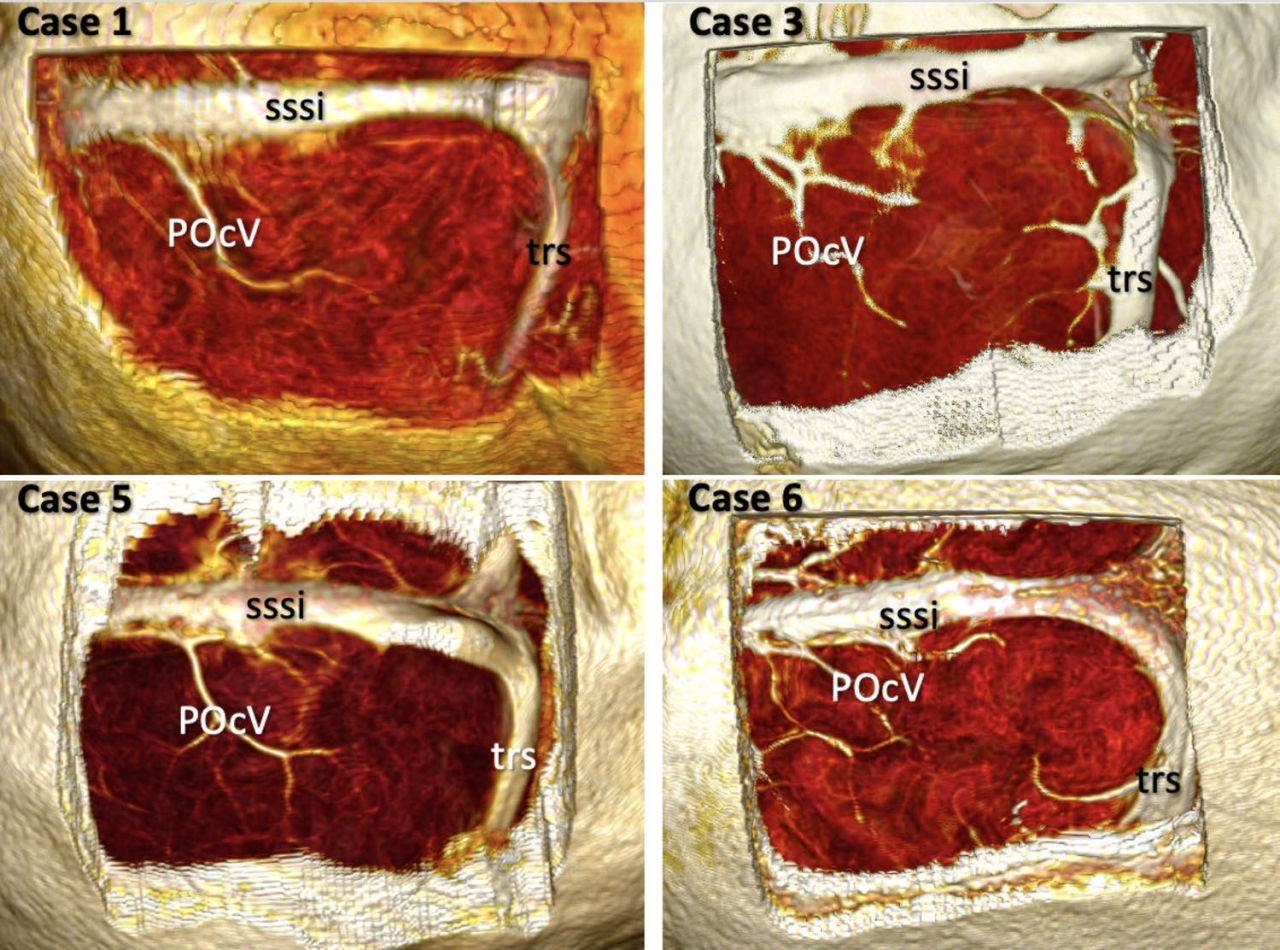
Three-dimensional MR venography reconstruction obtained using OsiriX software. On preoperative MR venography, the presence of a cuneus vein variant is suggested by an associated parieto-occipital vein that appears thinner than expected. POcV: Parieto-occipital vein sssi: Superior sagittal sinus trs: Transverse sinus

According to the literature, the parieto-occipital vein is usually located below the lambda at the level of the parieto-occipital fissure, with [14, 40] no other vein typically present between it and the occipital pole [14, 18, 21, 23, 31, 40]. In our series, however, after identifying the parieto-occipital vein at the level of the parieto-occipital fissure, we observed an additional vein extending toward the cuneus in 12 patients, more frequently in left-sided PIAs than right-sided approaches. The reason for its higher prevalence on the left remains unclear. In some patients, a variant referred to as the cuneus vein may be observed when the parieto-occipital vein is thinner than expected (Fig. 5).

The cuneus vein extends from posterior to anterior, has a horizontal entry into the sinus or falx, and is not visible on surface venography. We observed that this cuneus vein exhibited three distinct variations. In some patients, it drained directly into the superior sagittal sinus, in which the cut technique could not be used, and the vein could be released only through arachnoid dissection. In other patients, it drained into the falx, allowing the use of the falx-cutting technique (Fig. 1). In one patient, we observed the cuneus vein draining into the straight sinus (Fig. 3D).

This technique is similar to the tentorial cut technique we previously described [2]. Careful attention is needed during dissection to avoid compromising the cuneus vein, as inadvertent damage may lead to hemianopsia. We also observed that the parieto-occipital vein was considerably smaller in diameter when the cuneus vein was present, which we attributed to both veins draining the same region. We identified the cuneus vein in 12 of 303 PIAs and successfully preserved it in all but one patient. No evidence of hemorrhage or stroke-related morbidity was observed in the long-term follow-up of this patient.

The striate cortex, also known as the primary visual cortex, is situated within the medial occipital lobe, comprising the cuneus and lingual gyrus, and extends along the superior and inferior aspects of the calcarine fissure. The striate cortex and extra-striate visual association cortex both play critical roles in visual perception [4, 42]. Therefore, failure to preserve this variant cortical vein draining the cuneus can lead to temporary or permanent visual impairments, ranging in severity from hemianopsia to complete visual loss. Numerous studies report rates of permanent visual field loss ranging from 0 to 17% after surgery in this region [16, 25, 36].

Optimal anesthesia management and cerebrospinal fluid drainage via the subsplenial region are also critical to facilitate brain relaxation during the PIA. We take care to avoid placing retractors on the calcarine fissure to prevent inadvertent damage, and we aim to preserve the internal occipital and parieto-occipital veins to maintain vascular integrity. Excessive brain relaxation after cerebrospinal fluid drainage may predispose to vein tearing. Careful mobilization and release of the vein are therefore essential to prevent such complications. Special attention is given to the potential presence of the cuneus vein, particularly when the parieto-occipital vein appears thin, and we consider incising the falx or opening the arachnoid membranes if necessary (Video 1).

Venous sacrifice in neurosurgery remains a controversial and incompletely predictable maneuver, as cerebral veins lack the redundancy and collateralization typically observed in arterial circulation [20]. While some veins may appear dispensable intraoperatively, their interruption can result in impaired venous outflow, venous congestion, and subsequent increases in capillary hydrostatic pressure, ultimately leading to vasogenic edema, venous infarction, or hemorrhagic transformation [1]. The clinical consequences of venous injury are often delayed and may not be immediately apparent during surgery, further complicating intraoperative decision-making. A classic example of this controversy is the sacrifice of Dandy’s vein during approaches to the petroclival region, which has been reported as both clinically tolerated and, in other cases, associated with devastating venous infarction [10, 29, 30]. This variability underscores the importance of venous dominance, drainage territory, and the presence or absence of effective collateral pathways. Consequently, increasing emphasis has been placed on venous preservation whenever feasible, particularly for veins draining eloquent cortical or deep venous territories. In this context, unrecognized or underestimated venous variants—such as those described in the present study—pose a significant risk, as their inadvertent injury may disrupt critical drainage pathways and result in avoidable morbidity.

The PIA stands as a vital surgical approach to lesions in challenging locations. According to the literature, there are no veins in the cuneus or occipital regions other than the parieto-occipital vein, and a free space extends from the parieto-occipital fissure to the occipital pole. Accessing this area and cutting the tentorium provides a wide working space. However, when the cuneus vein is present, this approach could pose significant challenges. In such cases, we used arachnoid-releasing and falx-cutting techniques to preserve the vein. Preserving the cortical veins is paramount to minimizing complications and improving patient outcomes. Thus, it is essential to recognize that the cuneus vein may be encountered during the PIA, and it is crucial to protect it.

A relevant limitation of this study is the inability to reliably visualize the cuneus vein on preoperative MRI, which constrains its integration into surgical planning. This finding underscores the need for improved imaging techniques or adjunct modalities to enhance the preoperative delineation of small venous structures.

## Conclusion

This study identifies a previously underrecognized cortical venous variation arising from the cuneus and coursing toward the falx cerebri that may be encountered during the posterior interhemispheric approach. As this vein is not detectable on preoperative imaging, intraoperative recognition and preservation are essential to avoid venous problems and related morbidity. The falx-cutting and vein-releasing techniques described provide practical strategies for safely managing this variation. Awareness of this anatomy may enhance surgical safety within the posterior interhemispheric corridor.

## Supplementary Information

Below is the link to the electronic supplementary material.

ESM 1 Supplementary Material 1: Surgical demonstration of the cuneus vein in four patients including a description of the falx-cutting and vein-releasing technique. (MOV 193 MB)

## Data Availability

Not applicable.

## Declarations

### Declaration of Helsinki

The study was conducted in accordance with the declaration of Helsinki.

## References

[1] Anichini G, Iqbal M, Rafiq NM, Ironside JW, Kamel M (2016) Sacrificing the superior petrosal vein during microvascular decompression. Is it safe? Learning the hard way. Case report and review of literature. Surg Neurol Int 7(Suppl 14):S415–S420. 10.4103/2152-7806.183520 27313970 10.4103/2152-7806.183520 PMC4901823

[2] Berker BB, Dogruel Y, Gungor A et al (2024) Preserving the cerebellar hemispheric tentorial bridging veins through a novel tentorial cut technique for supracerebellar approaches. J Neurosurg 140(1):260–270. 10.3171/2023.5.JNS23657 37486872 10.3171/2023.5.JNS23657

[3] Chi JH, Lawton MT (2006) Posterior interhemispheric approach: surgical technique, application to vascular lesions, and benefits of gravity retraction. Neurosurgery 59(1 Suppl 1):41–49. 10.1227/01.Neu.0000219880.66309.85 10.1227/01.NEU.0000219880.66309.85 16888550

[4] Choi SH, Jeong G, Kim YB, Cho ZH (2020) Proposal for human visual pathway in the extrastriate cortex by fiber tracking method using diffusion-weighted MRI. Neuroimage 220:117145. 10.1016/j.neuroimage.2020.117145 32650055 10.1016/j.neuroimage.2020.117145

[5] Choque-Velasquez J, Hernesniemi J (2018) One burr-hole craniotomy: Posterior interhemispheric approach in Helsinki Neurosurgery. Surg Neurol Int 9:183. 10.4103/sni.sni_202_18 30283716 10.4103/sni.sni_202_18 PMC6157042

[6] Doğruel Y, Rahmanov S, Güngör A, Türe U (2023) Posterior interhemispheric transtentorial subsplenial approach for posterior thalamic tumors: 2-dimensional operative video. Oper Neurosurg. 10.1227/ons.0000000000000938 37811933 10.1227/ons.0000000000000938

[7] Elarjani T, Khan NR, Sur S, Morcos JJ (2021) Occipital posterior interhemispheric supratentorial approach for resection of midbrain cavernous malformation. Neurosurgical Focus: Video 2021(1):6. 10.3171/2021.4.FOCVID2133 10.3171/2021.4.FOCVID2133 PMC9549983 36284906

[8] Germann AM, Kashyap V. Anatomy, Head and Neck, Occipital Bone, Artery, Vein, and Nerve. 2023 Jul 24. In: StatPearls [Internet]. Treasure Island (FL): StatPearls Publishing; 2026 Jan–. PMID: 31082137. 31082137

[9] Gungor A, Gurses ME, Dogan E et al (2023) Interhemispheric Transcingulate Sulcus Approach to Deep-Seated Medial Frontal and Parietal Lesions-Fiber Dissection Study With Illustrative Cases. Oper Neurosurg (Hagerstown) 24(3):e178–e186. 10.1227/ons.0000000000000499 10.1227/ons.0000000000000499 36701601

[10] Hafez A, Nader R, Al-Mefty O (2011) Preservation of the superior petrosal sinus during the petrosal approach. J Neurosurg 114(5):1294–1298. 10.3171/2010.6.JNS091461 20617877 10.3171/2010.6.JNS091461

[11] Hakuba, A. (Ed.). (1996). Surgery of the intracranial venous system: Embryology, anatomy, pathophysiology, neuroradiology, diagnosis, treatment. Tokyo: Springer.

[12] Harput MV, Gonzalez-Lopez P, Türe U (2014) Three-dimensional reconstruction of the topographical cerebral surface anatomy for presurgical planning with free OsiriX software. Neurosurgery 10(3):426–435. 10.1227/neu.0000000000000355 24662508 10.1227/NEU.0000000000000355

[13] Karatas D, Martínez Santos JL, Uygur S et al (Jun.2023) A new classification of parasagittal bridging veins based on their configurations and drainage routes pertinent to interhemispheric approaches: a surgical anatomical study. J Neurosurg 01(2023):1–11. 10.3171/2023.4.JNS222866 10.3171/2023.4.JNS222866 PMC10238814 37310056

[14] Krayenbühl H, Huber P, Yaşargil MG. Cerebral angiography. Stuttgart: Georg Thieme Verlag; 1982.

[15] Krogager ME, Jespersen B, Mathiesen TI, Benndorf G (2022) Three underdogs among galenic veins: anatomical analysis and literature review of surgical relevant veins in the quadrigeminal cistern. Neurosurg Rev 45(5):3245–3258. 10.1007/s10143-022-01842-z 35947231 10.1007/s10143-022-01842-z

[16] Kurokawa Y, Uede T, Hashi K (1999) Operative approach to mediosuperior cerebellar tumors: occipital interhemispheric transtentorial approach. Surg Neurol 51(4):421–425. 10.1016/s0090-3019(98)00123-2 10199296 10.1016/s0090-3019(98)00123-2

[17] Kyoshima K, Oikawa S, Kobayashi S (2001) Preservation of large bridging veins of the cranial base: technical note. Neurosurgery 48(2):447–449. 10.1097/00006123-200102000-00047 11220394 10.1097/00006123-200102000-00047

[18] Lasjaunias P, Berenstein A, Brugge KT (2001) Clinical vascular anatomy and variations. Springer Berlin Heidelberg

[19] Nanda A, Konar S, Kalakoti P, Maiti T (2016) Posterior interhemispheric approach and microsurgical resection of a pineal parenchymal neoplasm of intermediate differentiation. Neurosurg Focus 40(Suppl 1):2016.1.FocusVid.15439. 10.3171/2016.1.FocusVid.15439 26722676 10.3171/2016.1.FocusVid.15439

[20] Narayan V, Savardekar AR, Patra DP et al (2018) Safety profile of superior petrosal vein (the vein of Dandy) sacrifice in neurosurgical procedures: a systematic review. Neurosurg Focus 45(1):E3. 10.3171/2018.4.FOCUS18133 29961377 10.3171/2018.4.FOCUS18133

[21] Newton, T. H., & Potts, D. G. (1971). Radiology of the skull and brain: Angiography (Vols. 1–4). St. Louis: Mosby.

[22] Oka K, Rhoton AL Jr., Barry M, Rodriguez R (1985) Microsurgical anatomy of the superficial veins of the cerebrum. Neurosurgery 17(5):711–48. 10.1227/00006123-198511000-00003 4069326 10.1227/00006123-198511000-00003

[23] Osborn AG, Hedlund GL, Salzman KL. Osborn’s brain: Imaging, pathology, and anatomy. Philadelphia: Elsevier Health Sciences; 2017.

[24] Panteli A, Gungor A, Firat Z, Saritepe F, Ture H, Ture U (2022) The posterior interhemispheric transparieto-occipital fissure approach to the atrium of the lateral ventricle: a fiber microdissection study with case series. Neurosurg Rev 45(2):1663–1674. 10.1007/s10143-021-01693-0 34822014 10.1007/s10143-021-01693-0

[25] Papo I, Salvolini U (1974) Meningiomas of the free margin of the tentorium developing in the pineal region. Neuroradiology 7(4):237–243. 10.1007/bf00342704 4414635 10.1007/BF00342704

[26] Rahmanov S, Doğruel Y, Güngör A, Türe U (2023) Contralateral posterior interhemispheric transtentorial suprapineal approach to the 3rd ventricle surface of the thalamus: 2-dimensional operative video. Oper Neurosurg 25(5):e289. 10.1227/ons.0000000000000839 37534891 10.1227/ons.0000000000000839

[27] Rhoton AL Jr. Rhoton: Cranial anatomy and surgical approaches. Philadelphia: Lippincott Williams & Wilkins (Wolters Kluwer Health); 2003.

[28] Ribas GC, Yasuda A, Ribas EC, Nishikuni K, Rodrigues AJ, Jr (2006) Surgical anatomy of microneurosurgical sulcal key points. Neurosurgery 59(4 Suppl 2):ONS177–210; discussion ONS210–1. 10.1227/01.NEU.0000240682.28616.b2 10.1227/01.NEU.0000240682.28616.b2 17041489

[29] Rosenblum JS, Neto M, Essayed WI et al (2019) Tentorial venous anatomy: cadaveric and radiographic study with discussion of origin and surgical significance. World Neurosurg 131:e38–e45. 10.1016/j.wneu.2019.06.232 31295599 10.1016/j.wneu.2019.06.232 PMC6819248

[30] Sakata K, Al-Mefty O, Yamamoto I (2000) Venous consideration in petrosal approach: microsurgical anatomy of the temporal bridging vein. Neurosurgery 47(1):153–160. 10.1097/00006123-200007000-00032 10917358 10.1097/00006123-200007000-00032

[31] Salamon G, Huang YP (1976) Radiologic Anatomy of the Brain. Springer-Verlag

[32] Serra C, Ture U (2022) The extreme anterior interhemispheric transcallosal approach for pure aqueduct tumors: surgical technique and case series. Neurosurg Rev 45(1):499–505. 10.1007/s10143-021-01555-9 33945071 10.1007/s10143-021-01555-9

[33] Serra C, Ture H, Yaltirik CK, Harput MV, Ture U (2020) Microneurosurgical removal of thalamic lesions: surgical results and considerations from a large, single-surgeon consecutive series. J Neurosurg 1–11. 10.3171/2020.6.JNS20524 10.3171/2020.6.JNS20524 33007756

[34] Serra C, Ture H, Firat Z et al (2023) Microsurgical management of midbrain gliomas: surgical results and long-term outcome in a large, single-surgeon, consecutive series. J Neurosurg 1–12. 10.3171/2023.5.JNS222219 10.3171/2023.5.JNS222219 37503951

[35] Serrano R, Babin E, Amor MB, Megret M (1972) Radio-anatomy of the internal occipital veins. Neuroradiology 3(3):153–154. 10.1007/BF00341499 4670604 10.1007/BF00341499

[36] Shirane R, Shamoto H, Umezawa K et al (1999) Surgical treatment of pineal region tumours through the occipital transtentorial approach: evaluation of the effectiveness of intra-operative micro-endoscopy combined with neuronavigation. Acta Neurochir (Wien) 141(8):801–8. 10.1007/s007010050380 10536715 10.1007/s007010050380

[37] Srinivasan VM, Catapano JS, Sheehy JP, Labib MA, Lawton MT (2021) Posterior interhemispheric occipital transtentorial approach for resection of a falcotentorial meningioma. Neurosurg Focus Video 5(1):V2. 10.3171/2021.4.Focvid2125 36284904 10.3171/2021.4.FOCVID2125 PMC9549996

[38] Sugita K, Kobayashi S, Yokoo A (1982) Preservation of large bridging veins during brain retraction. Technical note. J Neurosurg 57(6):856–858. 10.3171/jns.1982.57.6.0856 7143075 10.3171/jns.1982.57.6.0856

[39] Ture U, Yasargil MG, Al-Mefty O (1997) The transcallosal-transforaminal approach to the third ventricle with regard to the venous variations in this region. J Neurosurg 87(5):706–715. 10.3171/jns.1997.87.5.0706 9347979 10.3171/jns.1997.87.5.0706

[40] Yaşargil MG. Microneurosurgery. Vol. III B: AVM of the brain, clinical considerations, general and special operative techniques, surgical results, nonoperated cases, cavernous and venous angiomas, neuroanesthesia. Stuttgart: Georg Thieme Verlag; 1988.

[41] Yaşargil MG. Microneurosurgery. Vol. IV B: Microneurosurgery of CNS tumors. Stuttgart: Georg Thieme Verlag; 1995.

[42] Yoshimoto K, Araki Y, Amano T, Matsumoto K, Nakamizo A, Sasaki T (2013) Clinical features and pathophysiological mechanism of the hemianoptic complication after the occipital transtentorial approach. Clin Neurol Neurosurg 115(8):1250–1256. 10.1016/j.clineuro.2012.11.024 23260765 10.1016/j.clineuro.2012.11.024

